# Leveraging microgrants to support capacity-building workshops in low- and middle-income countries for meeting the Sustainable Development Goals

**DOI:** 10.5195/jmla.2022.1334

**Published:** 2022-01-01

**Authors:** Karin J. Saric, Masimba Muziringa, Erin R. B. Eldermire, Sarah Young, Israel M. Dabengwa

**Affiliations:** 1 ksaric@usc.edu, Norris Medical Library. University of Southern California. Los Angeles. CA; 2 mmuziringa@gmail.com, College of Health Sciences. University of Zimbabwe. Harare. ZI; 3 erb29@cornell.edu, Flower-Sprecher Veterinary Library. Cornell University. Ithaca. NY; 4 sarahy@andrew.cmu.edu, Carnegie Mellon University. Pittsburgh. PA; 5 israel.dabengwa@nust.ac.zw, Faculty of Medicine Library. National University of Science and Technology. Bulawayo. ZI

**Keywords:** microgrants, capacity-building, Africa, low- and middle-income countries, LMICs, global collaboration, medical library, medical librarian, Research4Life, Hinari, systematic review, synthesis research, evidence-based practice, information access, knowledge translation, Sustainable Development Goals, SDGs

## Abstract

Twenty fifteen marked the year of assessment for the Millennium Development Goals (MDGs). The MDGs that achieved the greatest success were those where evidence-based practice (EBP) interventions were implemented. The ability to practice evidence-based medicine is grounded in the creation of and access to medical literature that synthesizes research findings. The role that global health literature played in the success of the MDGs demonstrates that medical libraries and librarians have a role to play in achieving the Sustainable Development Goals (SDGs).

Librarians can hold capacity-building workshops that provide instruction on how to access evidence-based literature and also train health professionals to conduct synthesis research. Research findings conducted by in-country health professionals are more likely to address issues being faced by local communities and will afford the possibility of obtaining the necessary evidence-based answers that can then be used to implement policies to resolve public health issues identified in the SDGs.

This paper discusses how an international team of librarians leveraged funding from a Medical Library Association/Librarians without Borders/Elsevier Foundation/Research4Life grant to hold a capacity-building workshop in Zimbabwe and follow-up online trainings. The workshop focused on accessing evidence-based resources and conducting synthesis research. Outcomes included the creation and policy implementation of evidence-based knowledge products in alignment with local needs and galvanizing a multisectoral group of key individuals who have gone on to collaborate toward the vision of creating a Zimbabwe innovation hub. Looking ahead, such grants can be leveraged to conduct capacity-building to support knowledge translation and other local training needs.

## INTRODUCTION

Twenty fifteen marked the year of assessment for the United Nations' Millennium Development Goals (MDGs). Overall assessment demonstrated that the MDGs that achieved the greatest success were those where evidence-based practice (EBP) interventions were implemented [13]. Although access to literature has improved, Global North researchers still generate much of the global health literature, and this research is often not relevant to issues faced by low- and middle-income countries (LMICs) [4,5]. African researchers [6], African health leaders [7], and global health leaders from the North [8] have noted that health care in African countries begins in the home, is primarily provided by mothers, and the assistance of health care professionals is sought only when necessary. Due to the unique context-specific challenges that each community must address, “every country needs to cultivate and grow a critical mass of individuals, groups, and institutions that interact regularly among themselves and with their governments, parliaments, and civil society as agents of change” [7].

As transition begins to the Sustainable Development Goals (SDGs), persistent obstacles to tackle to ensure successful transfer of knowledge into evidence and practice for LMICs include 1) increasing primary research and synthesis studies specific to health conditions in the developing world, 2) ensuring findings from such studies are presented to policy makers in an understandable manner, and 3) integrating findings into policy and practice in alignment with the sociopolitical and cultural contexts of each country and local communities [4, 5, 7, 9, 10]. The ability to practice evidence-based medicine is grounded in the creation of and access to clinical trial findings and medical literature that further synthesizes research findings. The role that global health literature played in the success of the MDGs demonstrates that medical libraries and librarians have a strong role to play in addressing the specific challenges of knowledge generation and dissemination necessary to achieving the SDGs.

The triple helix of innovation is a framework grounded in the idea that “the interaction among university, industry, and government is the key to innovation and growth in a knowledge-based economy” [11]. Omaswa et al. note the central role of universities, research centers, and all institutions involved in knowledge creation and dissemination: “Africa will not transform without the active participation of these people and their institutions. Their ability to team up with both communities and politicians will be critical to Africa's future” [7]. African libraries are centrally positioned at the intersection of these three groups and already provide significant yet underrecognized support for local country development [12,13]. African health libraries are the nexus of the health environment—they are embedded within university systems, have direct ties to government institutions, and can collaborate with national library networks and public libraries throughout the local communities [4-7, 13, 14].

Below we discuss how five librarians from two Zimbabwean and three American institutions were able to leverage funding from a Medical Library Association/Librarians without Borders/Elsevier Foundation/Research4Life (MLA/LWB) grant to conduct a capacity-building workshop in Zimbabwe. The main goal of the workshop was to provide a framework for participants to develop skills in information retrieval and evidence synthesis, using information resources from Research4Life (R4L) programs. Furthermore, it also proved to be instrumental in generating knowledge products that have been implemented into policy in alignment with local needs, and it galvanized a multisectoral group of key individuals who have gone on to collaborate toward the national vision of creating a Zimbabwe innovation hub.

We begin with an overview of the development and evolution of R4L programs. We then discuss the implementation and impact of the Zimbabwe capacity-development workshop. We conclude by conceiving how future capacity-building workshops can support knowledge translation by developing local, networked information infrastructures in line with the triple helix of innovation framework to support evidence-based decision making to achieve the SDGs.

## RESEARCH4LIFE

### Development of a structural foundation for library capacity-building efforts in developing countries

Access to, and participation in, knowledge production and dissemination is recognized as a catalyst for development within the SDG agenda [4]. Established in 2002, R4L is a public-private partnership created to provide institutions in LMICs online access to high-quality, evidence-based, peer-reviewed content [15]. The program was developed with a holistic perspective that includes a multisectoral approach to address global development. Currently, R4L consists of five interconnected programs: Research for Health (Hinari), Research in Agriculture (AGORA), Research in the Environment (OARE), Research for Development and Innovation (ARDI), and Research for Global Justice (GOALI).

Beyond access to literature, R4L provides a central platform that connects LMICs, and core activities serve to address the tacit aspects of the research/knowledge production cycle that must be in place for relevant evidence to be produced and made accessible to all stakeholders. Over the last few decades, R4L has evolved into “a vital infrastructural input into the development of evidence-based policies and investment priorities that will maximise the chances of the SDGs being realized” [14]. This evolution can be broken into four phases that reflect how the programs have continuously developed in line with community growth and needs: Phase 1) access to information (P1), Phase 2) health information literacy and end-user training (P2), Phase 3) capacity-building (P3), and Phase 4) development of networks and collaborations (P4).

### Phase 1—access to information

R4L programs have been recognized as a catalyst to information access in LMICs [16–18]. Respondents to the 2014 Hinari impact survey acknowledged that use of the resource had improved their abilities to access research findings, conduct research with applicable in-country results, and improve their scientific writing skills [19]. By 2018, Hinari's collections had grown to include a large number of high-quality resources, sufficient to be recognized as a resource that is able to support medical education programs [17,20]. Despite the overall success and developments, it is still not a perfect solution. Even in 2020, the Research4Life 2020 User Review findings concluded that there is great value to those who use the resource; however, there is an ongoing need to generate broader awareness and provide ongoing training and support [21].

### Phase 2—end-user training/health information literacy

Resources can only add value if end-users are aware of their existence and have the skill set to navigate and extract relevant information from them. Early on, R4L established partnerships with key institutions such as the Medical Library Association (MLA), the Elsevier Foundation, and the Information Training and Outreach Centre for Africa (ITOCA) to develop Training of Trainers (TOT) programs. In addition to generating awareness and training end-users on resource use, initial workshops focused on developing health information literacy, for which there is a continued need as it plays a role in the achievement of SDG-3 [13]. Over the years, workshop content has also evolved to include all aspects of the research cycle, including modules on authorship to support inclusion of LMIC research findings in the global health literature corpus [22]. Additionally, through the R4L and ITOCA listservs, relevant workshops held by other institutions and programs are regularly promoted, thereby greatly increasing educational opportunities.

### Phase 3—capacity-building

In 2016, the MLA/LWB initiative was expanded to include microgrants [23]. The grants are broad in scope, thereby allowing for trainings to include capacity-building workshops tailored to address the local needs of specific communities. This can include development of skills for conducting synthesis research, use of geographical information tools, or workshops that focus on data management, access, and use. The workshops also serve to connect multisectoral stakeholders, thereby generating the social infrastructure and/or networks required for knowledge translation (KT)—the dissemination of information to proper channels to increase the integration of evidence into policy and practice [24].

### Phase 4—global networks/collaborations

Cooperation and partnerships have long been a vital aspect of health information librarianship, playing a role in sharing resources, providing services, and advocating for unmet needs and issues [25]. Global networks allow the possibility to leverage resources, scale knowledge, and quickly disseminate information electronically to successfully address global health issues. R4L has provided a stable platform for LMIC global communications. From this central hub, LMICs can network and interact with other organizations working toward the same goals (e.g., triple helix organizations, African EBP networks, Association for Health Information and Libraries in Africa, International Federation of Library Associations, World Health Organization (WHO) etc.).

To meet the SDGs, all regions of the world must move the needle to be inclusive of the entire spectrum of phases, from P1 (access to information) to P4 (global collaborations). The establishment of MLA/LWB's microgrants has provided the opportunity to generate ground-level support for capacity-development in three key ways: 1) they support development of a community of trainers that can conduct trainings beyond university settings, 2) they allow latitude for tailored instruction on topics that address local needs, and 3) they are instrumental in galvanizing key individuals from a variety of backgrounds, which is a key element of moving communities into P4.

## THE WORKSHOP/S

Research by Popoola et al. identified a training need for librarians in Nigeria regarding EBP and systematic review (SR) [26]. Moreover, a 2017 survey to assess evidence synthesis capacity in Africa found that the top two reported hindrances to conducting evidence syntheses were “capacity, including experience, networking and collaboration” and “access to research literature and data” [27]. Having recently held independent end-user trainings (P2) in various African countries that focused on information retrieval and conducting evidence syntheses, the authors recognized that a capacity-building workshop that combined these two goals offered a more cohesive learning experience, as database searching for EBP literature is a required skill set for conducting SRs.

In 2019, the authors were awarded an MLA/LWB grant, which supported the development of two interconnected workshops—Workshop 1: *Accessing evidence-based resources via Research4Life* (two days) and Workshop 2: *Introduction to conducting systematic reviews, publishing and author resources* (three days). The aim of these trainings was to address gaps in knowledge about access to critical information resources for research and EBP and to lay the foundational skills for conducting evidence synthesis using systematic methods.

The two in-person trainings were held in July 2019 at the University of Zimbabwe (UoZ) Library. The trainings were free and open to all researchers, clinicians, faculty, students, and library staff, as well as individuals working in the public sector in government agencies and international government organizations. Broad outreach to R4L countries in Africa resulted in seventy-six applicants from a variety of disciplines from Southern and Eastern Africa. Applicants had the option to register for either one or both workshops with the large majority registering for both. Due to limited space, we chose to prioritize those based at Zimbabwean institutions and admitted forty-five participants including researchers, clinicians, ministry staff, and librarians. Ultimately, an average of thirty-seven participants from eight institutions attended all five days of the workshops.

### Workshop content

The workshops followed the traditional R4L TOT format that includes a combination of lectures followed by considerable hands-on exercises and group work, thereby actively applying learned content to practice in context.

Workshop 1 included an overview of the EBP process, as well as a deep dive into the Hinari research portal. Relevant content from AGORA and OARE were also highlighted. The content emphasized development of searching skills for EBP literature in several databases, as well as free online health resources. All instructional content was tailored to emphasize database content and features that support the creation of synthesis research.

Workshop 2 provided an introduction to SRs and in-depth training in problem formulation, eligibility criteria, and systematic searching. The authorship module included instruction on citation management and information on publishing from an author's' perspective. A full agenda is available at [28]. All workshop materials were made available to participants via Google Drive, such that they can serve as an ongoing resource beyond the workshop [29].

### Synthesis research support

Based on our past experience teaching SR methods, we understood that teaching this topic without immediate application of the skills or without establishing ongoing support could hinder long-term learning and impact. With this in mind, we designed the SR training to apply learned skills in the context of developing an SR protocol with topics developed by participants in advance of the training. Participants were encouraged to enroll in the program with potential collaborators, including librarians, with the aim of establishing project teams that would carry out SRs beyond the timeframe of the training. For this reason, the focus of the in-person, three-day training was on early-phase aspects of the SR process and protocol development.

To support later stages of the projects, a series of webinars was offered synchronously but recorded for later viewing over the following year. These webinars addressed topics such as translating search strategies across databases and using study screening software. A webinar on assessing study quality and risk of bias was also offered and was cotaught by one of the workshop participants, a biostatistician with expertise in research methods. We also offered monthly, remote, drop-in office hours for participants to ask questions related to their ongoing work or other SR projects in which they were involved.

### Workshop survey results/impact

**Figure 1:**
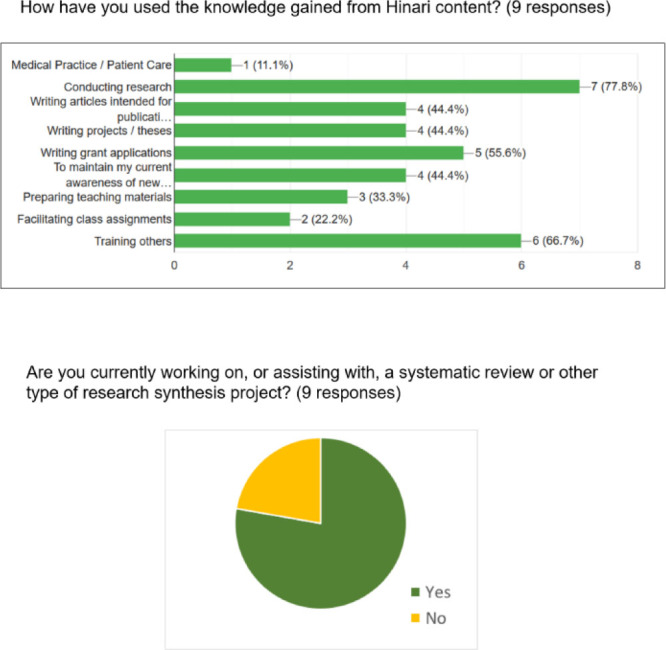
Survey results—six months postworkshop

The participants in both workshops were highly engaged, and postworkshop assessments indicated substantial impact. In a survey conducted immediately following the workshop, feedback was overwhelmingly positive, with participants indicating that the content was highly relevant and the format effective and engaging. Attendance at the follow-up webinars and online office hours was rather low, possibly due to scheduling challenges given the time zone differences. However, recorded webinars were viewed asynchronously, and several participants noted their usefulness as a resource for ongoing training. A follow-up survey was also conducted six months after the in-person training. Nine participants responded, of which 78% (n=7) indicated that they had used the knowledge gained from the first workshop on Hinari in their conduct of research, and 67% (n=6) specified that they had trained others on the use of Hinari. Regarding the SR training, 75% of respondents noted that they were currently working on or assisting with an SR six months after the training (n=9). At least one of those resulted in part from connections made at the workshop [30]. See Figure 1 for additional survey results.

Beyond skills training and SR production, other impacts have been noted. The workshops enabled UoZ's 2019–2025 strategic plan [31], in particular, by directly supporting Zimbabwe's national initiative Education 5.0 [32], which promotes the development of key skill sets to develop the workforce. They also supported institutional-level goals to develop global collaborations. However, the most significant impact was the ability to affect national policy. The training was held at a time when UoZ was working with the Ministry of Health and Child Care (MOHCC) in embedding the use of research evidence in policy and practice. Both university and ministry staff attended the training. As a result of this cross-institutional collaboration, seven policy products have been developed and implemented in relation to local needs and issues. See Table 1 for a sample subset. Two years later, this collaboration continues in a sustainable manner with the librarian participating in MOHCC meetings and assisting in addressing information needs. Finally, an unforeseen outcome was that the workshop served to galvanize key individuals from a variety of backgrounds who have continued to collaborate toward the national vision of creating a Zimbabwe innovation hub.

**Table 1:** Knowledge outcomes

Rapid review question and date	Rapid review objective	Status on implementation
1. What is the enrollment criteria and contributions of the informal sector into the national health insurance within the context of Zimbabwe? [October 2019]	The Government of Zimbabwe (GoZ) is working toward achieving universal health coverage by establishing an integrated health system that will increase available resources for health, reduce the burden of out-of-pocket expenditures for households, and promote efficiency and equity in the use of resources for health.	A policy position has been drafted. The draft policy is undergoing the necessary legislative procedures before expected implementation.
2. What is the effectiveness of use of nonsurgical face masks by the public/community for effective infection prevention and control of COVID-19 in low resource settings? [April 2020]	The GoZ considered the use of face masks by the general public as a complementary safety measure to local and community transmission, especially in all public spaces such as markets, grocery stores, shopping centers, when using public transport, at funerals, and in work spaces.	The policy on compulsory wearing of facemasks was approved and implemented in Zimbabwe on 16 April 2020 for infection prevention and control of COVID-19.
3. What is the effectiveness of hydroxychloroquine/chloroquine in the hospital management of COVID-19 patients? [11 August 2020]	In the absence of vaccines approved [August 2020], the GoZ sought to establish whether the immunomodulatory effect of hydroxychloroquine may be useful in controlling the cytokine storm that occurs late phase in critically ill patients with SARS-CoV-2.	There was a paucity of evidence on the efficacy of hydroxychloroquine in hospital management of COVID-19 patients. The health care professionals and the community were discouraged from using the drug by the GoZ.

Overall, the program addressed key pillars of research capacity-building [33] in line with the triple helix of innovation through the application of skills to practice in an evidence synthesis context, building collaborations between key stakeholders, impacting policy, and planting seeds for the development of strategic institutional partnerships. Furthermore, as instructors, we each learned important lessons from each other that have deepened our knowledge and situational understanding across global contexts.

## DISCUSSION

We cannot draw conclusions regarding the impact of this workshop on achieving the SDGs; however, this collaboration has demonstrated that librarians can support access to evidence-based literature, promotion of health literacy, creation of local knowledge, implementation of evidence into policy, and development of a networked infrastructure that can work toward bridging the communication gaps between key institutions identified in the triple helix model (university, industry, and government).

With the strong interest in synthesis research in LMICs [27,34], the generation of local evidence is likely to increase. Moving forward, librarians can continue to leverage workshop grants to support the next natural step, integrated knowledge translation (IKT), which relies on stakeholders' coproduction of knowledge for decision-making. IKT has been shown to improve the implementation of evidence into policy and practice as it allows actors to collaboratively identify high-priority questions, gaps in knowledge, and synthesize and disseminate findings [35].

To build IKT capacity, professionals need to be educated in the fundamentals and application of KT [24,35]. Although best done at the organizational level [36], there is a lack of infrastructure to support such collaborative efforts [27,35]. Research suggests that workshops can be effective in filling this gap [37]. R4L workshops inherently provide the necessary collaborative structure and can serve to create communities of practice and information networks around high-priority issues [38]. Librarians can serve as neutral knowledge brokers to connect key stakeholders, provide foundational KT education, drive innovative ways of creating evidence, and facilitate knowledge exchange [36,39].

## CONCLUSION

Librarians can leverage small- and medium-sized grants, like the MLA/LWB microgrants, to hold capacity-building workshops to break down barriers around access to, generation of, and dissemination of scientific knowledge required to achieve the SDGs. Workshops can focus on developing skills around database searching, evidence synthesis creation, KT, and more. Moreover, the workshops provide the opportunity to develop multisectoral networks to collectively work toward resolution of regionally relevant, high-priority issues. As part of such multisectoral teams, librarians have the ability to function as discipline-neutral knowledge brokers that can facilitate understanding of barriers faced by actors within triple helix institutions (university, industry, and government). Overall, librarian-specific skills of training, finding, analyzing, synthesizing, organizing, storing, and disseminating high-quality evidence can contribute to reducing the knowledge translation gap by implementing research findings into policy and practice in a timely manner to resolve global health issues.
